# A survey of the effective factors in students' adherence to university dress code policy, using the theory of reasoned action

**Published:** 2015-07

**Authors:** MOHAMMAD HOSSEIN KAVEH, LEILA MORADI, MARYAM HESAMPOUR, JAFAR HASAN ZADEH

**Affiliations:** 1 Department of Health Education and Promotion, School of Health, Shiraz University of Medical Sciences, Shiraz, Iran;; 2 Department of Epidemiology, School of Health, Shiraz University of Medical Sciences, Shiraz, Iran

**Keywords:** Dress code, Adherence, Behavior

## Abstract

**Introduction:**

Recognizing the determinants of behavior plays a major role in identification and application of effective strategies for encouraging individuals to follow the intended pattern of behavior. The present study aimed to analyze the university students’ behaviors regarding the amenability to dress code, using the theory of reasoned action (TRA).

**Methods:**

In this cross sectional study, 472 students were selected through multi-stage random sampling. The data were collected using a researcher-made questionnaire whose validity was confirmed by specialists. Besides, its reliability was confirmed by conducting a pilot study revealing Cronbach’s alpha coefficients of 0.93 for attitude, 0.83 for subjective norms, 0.94 for behavioral intention and 0.77 for behavior. The data were entered into the SPSS statistical software and analyzed using descriptive and inferential statistics (Mann-Whitney, correlation and regression analysis).

**Results:**

Based on the students’ self-reports, conformity of clothes to the university’s dress code was below the expected level in 28.87% of the female students and 28.55% of the male ones. The mean scores of attitude, subjective norms, and behavioral intention to comply with dress code policy were 28.78±10.08, 28.51±8.25 and 11.12±3.84, respectively. The students of different colleges were different from each other concerning TRA constructs. Yet, subjective norms played a more critical role in explaining the variance of dress code behavior among the students.

**Conclusion:**

Theory of reasoned action explained the students’ dress code behaviors relatively well. The study results suggest paying attention to appropriate approaches in educational, cultural activities, including promotion of student-teacher communication.

## Introduction

The way we dress is one of the main elements and manifestations of culture and is different in various societies (1). Clothes are not only used to physically protect the body, but they are also representative of a society’s culture as well as a strong communicative tool for transfer of inter-personal and social messages (2). In fact, clothes and the message they transfer are considered the main indicator of a society’s culture. Individuals may develop a positive or negative attitude toward a specific group of the society due to their way of dressing (3). Viewpoints, norms, and values are the particular dimensions of culture which are reflected in the individuals’ appearance. Thus, clothes usually provide the basis for formation of attitudes and immediate views towards the individuals who use them (4). Overall, way of dressing is important from two perspectives: 1- individual function: which includes responding to the innate need for covering oneself and supplying one’s safety against physical as well as chemical factors, 2- social function: one of the social functions of dressing is its identification capacity, because type of dressing represents a society’s traditions, values, and beliefs (5).

University, students and professors in particular, has a specific position among the public. In other words, people consider students and professors to be different from other organizations and social institutions and have different expectations from them (6). A large proportion of these effects occurs during confrontations, simple interactions, and observations. These seemingly simple observations have a great impact on the public’s judgments, attitudes, and patterns of behavior. Thus, elegance and dressing pattern of the academics are of great importance (7).

Universities in many countries of the world have attempted to define specific dress codes through various mechanisms, such as laying down laws and considering evaluation criteria(8). However, in spite of the emphasis on the issue and development of executive measures, evidence has shown that amenability to the defined dress code has been below the expected level. For instance, up to 2005, only 14% of the American schools followed dress codes(9).

Behavior is affected by a large number of intra-personal, inter-personal, and demographic factors (institutes, social culture, and policies) (10). Thus, adolescents’ way of dressing is affected by various factors, such as social status, education, media, and peer pressure (11). Therefore, identification and scientific analysis of behavior is highly important in planning educational and cultural interventions. In this regard, behavior change theories can help a great deal in more efficient analysis of dressing behavior. In addition, theory-based researches have facilitated comparison of the results obtained in different studies (12). One of the theories which seems to be beneficial in analyzing dressing behavior is the theory of reasoned action. This theory emphasizes behavioral intention as the most important determinant of behavior (13). Behavioral intention is defined as an individual’s motivation for conscious planning and one’s decision or intention for conducting the target behavior (14). According to the theory of reasoned action, behavioral intention is affected by two main determining factors, namely attitude toward the intended behavior and the subjective norms related to that behavior (13). Attitude has been defined as a person’s evaluation of desirability or undesirability of a behavior and its outcomes. For instance, in case a student believes following the university’s dress code to be beneficial in improvement of one’s position inside and outside the organization and commitment to the defined dress code causes no restrictions in his/her interactions and being prissy, that student will have a positive evaluation and will be interested in following that pattern (15).

Subjective norm refers to an individual’s perception of expectations of the individuals s/he considers important and effective regarding performance or avoidance of a particular behavior (15).

In general, way of dressing is not only affected by personal wants, interests, and preferences, but it is also influenced by real or perceived pressures of the social environment (16). Since adolescents’ and adults’ way of dressing is a major issue in most communities, social organizations, and educational institutions, performance of a theory-based research on this issue seems to be essential. Therefore, the present study aims to predict the students’ amenability to university’s dress code using the theory of reasoned action.

## Methods

In the present cross-sectional study, 472 students of Shiraz University of Medical Sciences who had studied in the university for at least a year and more than a semester was remained to their graduation took part in the study. Written informed consents for taking part in the study were obtained from all of the students. The demographic variables included sex, age and field of study.

The study data were collected using a researcher-made questionnaire including demographic variables, information about dress code, and constructs of the theory of reasoned action.

In order to measure the students’ information about dress code in the university, a question was posed including the following options: “No” (no information), “I have only heard its name”, “I have only read the generalities on the board”, “I have only heard the generalities from professors and students”, and “I have completely read the dress code content”. The options were scored as 0-4.

The constructs of the theory of reasoned action were attitude, subjective norms, behavioral intention, and behavior.

The students’ attitude toward dress code was evaluated using 6 questions. One of these items was “Execution of dress code in universities promotes a positive view of the universities in the society”. The attitude questions were responded through a 7-option Likert scale ranging from 1 (completely agree) to 7 (no idea). Finally, attitude scores ranged from 6 to 42. Higher scores represented a strong positive attitude, while lower scores showed a highly negative attitude toward dress code-based behavior.

The students’ behavioral intention toward following the university’s dress code inside and outside the university was assessed using two separate questions. These questions were responded through a Likert scale ranging from 1 (completely disagree) to 7 (completely agree). Score of 7 was assigned to the students who dressed in complete accordance with the university’s dress code. The minimum and maximum scores of this construct were 2 and 14, respectively.

Subjective norms were evaluated using 6 questions responded through a Likert scale ranging from 1 (completely disagree) to 7 (completely agree). Finally, subjective norms scores ranged from 6 to 42.One of these questions was “My family emphasizes my dressing to be in accordance with the society’s basic cultural values”. Higher scores represented stronger subjective norms toward following the appropriate dressing pattern considering the basic cultural values of the society.

In order to measure the students’ behavior, the university’s dress code, in the form of 11 items, was separately developed for male and female students. Then, the study participants were asked to evaluate their conformity to the dress code using a graphic rating scale (low, average, high, and complete concordance). Afterwards, these rates were scored from 1 (low concordance) to 4 (complete concordance) and in total, behavior scores ranged from 11 to 44. Finally, the two upper rates (high and complete) were considered as desirable, while the two lower ones were considered undesirable or below the expected level. 

The content validity of the questionnaire was confirmed by 5 experts. In order to determine the reliability of the questionnaire, a pilot study was conducted on 40 study students and Cronbach’s alpha coefficients of 0.93, 0.82, 0.94, and 0.77 were obtained for attitude, subjective norms, behavioral intention and behavior, respectively. 

The samples were selected through stratified proportional sampling. Shiraz University of Medical Sciences is the largest university in South of Iran and contains eight colleges. This university has the largest number of students from different parts of the country.

At first, the number of students studying in the 8 colleges of Shiraz University of Medical Sciences was determined. Then, the number of students in each college who were required to take part in the study was determined. The qualified students were identified based on the information in the Department of Education. In the colleges in which the number of qualified students was equal or close to the number of students who had to be selected from that college, all the students were enrolled into the study. In the colleges in which the number of students was more than the number required for the study, at first several classes were selected through simple random sampling and all the students of those classes were recruited into the study. Finally, the questionnaires were distributed among the students. It should be noted that the students were informed about the study procedures and objectives. They were also assured about their anonymity and that participation/non-participation in the study was voluntary. After all, more than 95% of the questionnaires were completely returned and the data underwent statistical analysis.

All the statistical analyses were performed using the SPSS software, version 14 (SPSS Inc., an, IBM Company). In order to describe the constructs of the theory, i.e. behavior, intention, attitude, and subjective norms, measures of central tendency and dispersion were employed. In addition, Mann-Whitney test was used to compare the frequency distribution of the students based on sex and the constructs of the theory. Besides, the mutual relationship among the constructs was assessed using Spearman correlation coefficient. Finally, regression analysis was used to determine the predictor variables of behavioral intention and behavior. The significance level was set at 0.05. 

## Results

The present study was conducted on 472 students of Shiraz University of Medical Sciences. Among the participants, 318 (67.4%) were female and 154 (32.6%) were male. Considering age distribution, 439 students (93%) were 18-23 years old, and the rest were 24-30 years old or above. Moreover, most of the study participants (163, 34.5%) belonged to the School of Medicine, 84 (17.7%) were studying in the School of Nursing and Midwifery, 49 (10.4%) belonged to the Paramedical School, and the rest were the students of other colleges.

According to the study results, only 18.6% of the students stated that they had read and were completely informed about the dress code. On the other hand, 11.4% of the students had no information about the dress code and the rest (70%) mentioned that they had only heard or read the generalities on the board.

Based on the students’ self-reports, conformity with the university’s dress code was below the expected level; only 28.87% of the female students and 28.55% of the male ones conformed to the dress code regulations. On the other hand, 71% of the students believed their way of dressing was highly in conformity with the dress code. The highest frequency of nonconformity among the female students was related to “wearing long and loose coats and pants and long scarf” (48.4%), “wearing coats with university’s logo and having photo identification cards” (39%), and “completely covering one’s hair and observing religious instructions” (32.4%). The highest frequency of such behaviors among the male students was related to “keeping one’s cell phone off in the class, conference room, and library” (53.2%), “wearing coats with university’s logo and having photo identification cards” (50.6%), and “avoiding chewing gums in the class and conference room in the presence of professors” (42.2%).

The descriptive statistics related to the constructs of theory of reasoned action are presented in Table 1.

**Table 1:** Descriptive statistics of the constructs of theory of reasoned action (n=472)

**Constructs**	**Mean** **±SD**	**Med**	**Min**	**Max**
**Behavior**	33.71±6.85	34	11	44
**Intention**	11.12±3.84	14	2	14
**Attitude**	28.78±10.08	31	6	42
**Subjective norm**	28.51±8.25	30	6	42

Considering behavioral intention, 294 students (62.3%) stated that their way of dressing was in accordance with the university’s dress code. However, a smaller number of students (almost 50%) mentioned that their way of dressing outside the university was in accordance with the dress code. Out of the 178 students whose dressing was not in accordance with the dress code, 41 (23%) mentioned that they intended to follow the dress code in future, 47 (26.4%) did not have such an intention, and 90 had no idea in this regard. Moreover, among the 238 students whose way of dressing out of the university was not in accordance with the dress code, 44 (18.5%) stated that they agreed to follow the university’s dress code outside the university, 85 (35.7%) were against this idea, and 109 (45.8%) had no clear opinion in this respect.

The findings of the present study revealed no significant difference between the students studying in different colleges and fields of study regarding the central tendency indexes of their dress code-related behavior. The lowest and highest mean scores of behavioral intention were related to the students of the College of Pharmacy (9.95+5.40) and School of Health (12.84+2.48), respectively. The medians of intention were respectively 10 and 14 in these two colleges (x(2)=10.73, p<0.001).

The lowest central tendency indexes of attitude were observed in the College of Pharmacy (Med-28), Dental School (Med-29), and Paramedical School (Med-29), while the highest ones were related to the School of Health (Med-35) and School of Management (Med-35) (x(2)=16.81, p=0.004).

Considering subjective norms, the lowest and highest medians were related to the Dental School (Med-25) and School of Health (Med-33), respectively (x(2)=16.81, P=0.019).

Considering the students’ self-reports, no significant difference was observed between the two sexes regarding their dress code-related behavior. However, the means and medians of behavioral intention, attitude, and subjective norms were significantly higher among the female students compared with the male ones (Table 2).

**Table 2:** Comparison of male and female students’ mean scores of knowledge,attitude, subjective norms and behavior toward dress code

**Sex**	Statistics	Behavior	Intention	Attitude	Subjective Norm
**Female(n=318)**	Mean±SD	33.57±7	11.43±3.66	29.68±9.96	29.18±7.95
	Med	34	14	32	31
**Male (n=154)**	Mean±SD	34.01±6.54	10.47±4.13	26.90±10.12	27.11±8.68
	Med	34.50	12	29	29
**Mann-Whitney test**		Z=-0.449 P=0.654	Z=-2.419 P=0.016	Z=-3.071 P=0.002	Z=-2.157 P=0.031

The correlation matrix of the study constructs is presented in Table 3. Accordingly, a significant positive correlation was observed among all the study constructs. In this regard, significant correlations were found between attitude and behavioral intention (r=0.571, p<0.01), attitude and subjective norms (r=0.638, p<0.01), attitude and behavior (r=0.327, p<0.01), subjective norms and intention (r=0.486, p<0.01), subjective norms and behavior (r=0.399, p<0.01), and behavioral intention and behavior (r=0.359, p<0.01).

**Table 3:** Correlation matrix (Spearman rho) among the study constructs in thestudy participants

**Constructs**	**Subjective norm**	**Behavioral intention**	**Behavior**
**Attitude**	0.638	0.571	0.327**
**Subjective norm**	-	0.486**	0.399**
**Behavioral intention **	-	-	0.359**

Note: Correlation is significant at*p<0.05, **p<0.01(2-tailed)

In order to predict the behavioral intention for following the dress code (dependent variable), attitude and subjective norms were assessed as independent variables using regression analysis (Table 4).Based on this model, attitude and subjective norms significantly (R(2)=0.442) accounted for the variance of the students’ intention (F(2,469)=185.48, p<0.001).

**Table 4:** Regression analysis by enter method for the predictor variables of the behavioral intention for dress code-related behavior

**Variable**	**B**	**SEB**	**β**	**t**	**p**
**Constant**	2.71	0.488	-	5.54	<0.001
**Attitude**	0.189	0.017	0.496	10.87	<0.001
**Subjective norm**	0.105	0.021	0.224	4.92	<0.001

R^2^=0.442, F(2, 469)=185.48, p<0.001

Regression analysis was also used to predict the dress code-related behavior (dependent variable) using attitude, subjective norms, and behavioral intention as independent variables (Table 5). The results showed that the prediction model including attitude, subjective norms, and intention was statistically significant (F(3, 468)=29.41, p<0.001) and explained 16% of the variance of the students’ behavior (R(2)=0.16). According to this model, most of the variance in dress code-related behavior was explained by behavioral intention followed by subjective norms. However, no significant role was observed for attitude in this model.

**Table 5:** Regression analysis by enter method for the predictor variables of the dress code-related behavior among the study participant

**Variable**	**B**	**SEB**	**β**	**t**	**p**
**Constant**	23.83	1.104	-	21.58	**-**
**Attitude**	0.001	0.043	0.002	0.030	0.97
**Subjective norm**	0.195	0.048	0.235	4.08	<0.001
**Behavioral attitude**	0.386	0.101	0.216	3.81	<0.001

R^2^=0.16, F(3, 468)=29.41, p<0.001

## Discussion

Elegance and way of dressing is of utmost importance among the academics. Dress code is applicable both inside an organization and out of the organization in the society (5). Therefore, professors’ and students’ dressing is considered as a major issue in most of the universities around the world, leading to development of laws and regulations in this regard (17).

Students’ low or no information about the university’s dress code suggests the necessity to review the information mechanisms as well as communication channels both qualitatively and quantitatively. The dominant methods of informing the students regarding dress code is through introduction of some guidelines in highly crowded meetings, such as inaugural programs, providing the students with a booklet or CD, sending the guidelines to colleges through bureaucratic channels, and putting the major points on bulletin boards. However, inter-personal relationships are more effective in information transfer as well as in change of behavior and attitude (12).

Based on the students’ self-reports in this study, and the students’ mean score of behavior, conformity with the university’s dress code was below the expected level. 

In contrast to the findings of the present study, the students’ knowledge level was high in the study conducted in Nigeria(18). Nevertheless, in spite of the university’s great attempts, including information transfer and lecturing, a considerable percentage of the students’ behavior was against the dress code.

Although self-report has been mentioned to be accompanied by several limitations, nonconformity of 29% of the students with the university dress code should not be ignored. It should be noted that the frequency of undesirable status was significantly higher than the above-mentioned measure in some items. It is also worth noting that these behaviors were observed in the students who been at least a year in the university. This suggests the necessity to review the mechanisms used in the university, such as development of guidelines, laying down laws, information, and cultural programs.

The findings of the current study revealed no significant difference between the two sexes as well as between the students studying in different colleges regarding their mean scores of behavior. This component might be more influenced by the students’ culture and social background. 

The findings of a study indicated that sex had no significant effects on following clothes behavior. They explained that this might be due to the fact that the individuals who aim to select and buy clothes go through a similar process of decision making irrespective of sex. The results of regression analysis in their study showed that selection and purchase of clothes were affected by cultural, economic, and personal variables, with social norms being the most important cultural variables (19).

In the same line, a study proposed that social norms were the main cultural factors affecting the type of dressing people choose(20).

These findings might also be due to the similar social atmosphere, such as induction of similar expectations for the two sexes. Consequently, students might also have similar perceptions regarding the expectations of the university’s social environment regarding their dressing method. These similar perceptions are, in turn, effective in conduction of similar responses and behaviors. Yet, longitudinal studies should be carried out to provide accurate answers to the following questions: “Is the university effective in modification or regulation of the students’ dressing behavior” and “To what extent is the university effective in this regard”. 

In spite of the fact that the present study participants’ behavioral intention towards dress code-based dressing was relatively good, it was still far from the desirable level. According to the results, only half of the students were serious in their intention to follow the university’s dress code (score of 14). In this respect, social desirability should be taken into account.

Analysis of the students’ responses to behavioral intention questions indicated that most of the students (77%) who considered their way of dressing to be in contrast to the university’s dress code either did not intend to change their behavior or had no specific idea in this regard. In addition, a large number of students disagreed to change their way of dressing out of the university. It should be mentioned that change of intention is quite different from change of knowledge and information level with respect to methods and difficulties. Thus, the authorities of the educational and cultural affairs should identify and apply appropriate educational and communicational methods in this regard.

The present study results demonstrated a significant difference between male and female students concerning their behavioral intention so that female students were more serious in changing their way of dressing inside and outside the university based on the university’s dress code. This difference in the students’ intention might be related to and affected by their attitude and subjective norms. These two constructs were also higher among the female students. The relatively strong correlation between these two constructs supports this claim, as well. Yet, it might also be associated with national religion and culture. In fact, attitude and particularly subjective norms are impacted by values, religious beliefs, and social culture (21).

In the present study, the students’ mean scores of attitude were far from the perfect level. The results showed that 26% of the students had evident negative attitudes towards the university’s dress code. The frequency of negative attitudes was also higher in some items. Since information transfer was quite limited among the students, more attempts should be made and specific measures should be taken to change their attitude towards this issue.

In a study performed in Nigeria also, a large number of respondents had a negative attitude towards the dress code and 65% believed this guideline was against their basic rights and freedom. Although 40% of the respondents believed that the dress code was effective in increasing the students’ desirable professional and social characteristics, 52% were opposed to this belief (18).

 The findings of the present study showed that the students’ subjective norms were considerably far from the desirable level. Besides, support of important individuals, such as parents, instructors, and peers, was important for 67% of the students. According to the study results, only 8% of the students were informed about the dress code through professors and other students. In spite of the importance of professors and peers for students, information transfer in this way was quite limited. Thus, new measures should be taken for improvement of information transfer through professors and other students.

The results of the present study indicated significant correlations between attitude and subjective norms as well as between behavioral intention and behavior. The average correlation between behavioral intention and behavior is in agreement with the claim that intention is the determinant of behavior in the theory of reasoned action (13). A meta-analysis conducted on the issue also demonstrated an average correlation between behavior and behavioral intention (14). These findings emphasize the role of other variables, such as perceived behavioral control, in determination of behavior (14). Most studies have also confirmed the role of social factors which is reflected in subjective norms (22).

In this study, a relatively strong correlation was found between subjective norms and intention. In addition, a moderate correlation was observed between subjective norms and behavior. A meta-analysis of several studies also demonstrated a moderate correlation between these two constructs (0.34) (14).

According to the theory of reasoned action, subjective norms affect behavior through intention (13). Additionally, peers are among the major social factors affecting subjective norms. Several studies have referred to the effect of peers on the development of behaviors such as drug abuse in adolescents (23) and dress code in girl adolescents (11). Yet, normative social influence is very strong and widespread. In addition to peers, parents, teachers, and sports dignitaries are among the sources of this influence (24).

The present study findings demonstrated a strong correlation between attitude and behavioral intention. The results of a meta-analysis also demonstrated a strong correlation between these two constructs (0.51) (14). Additionally, a large number of other studies have supported the strong correlation between attitude, behavioral intention, and behaviors such as driving(25), diabetes self-care (26), and interaction with a child (27).

The results of regression analysis in this study indicated that attitude and subjective norms significantly explained the variance in behavioral intention related to dress code. This implies that the participants with a more positive attitude and higher perceived social support regarding dressing in accordance with the university’s dress code have a higher tendency to dress according to this dress code. This finding is in agreement with the fundamental structural relationships of the theory of reasoned action. In this theory, the individuals’ attitude and subjective norms regarding a particular behavior are the main determinants of the behavioral intention (13). Nevertheless, the relative weight of these two constructs in the prediction model of behavioral intention might change based on the type of behavior or situation, such as characteristics of the population under study (14). The results obtained in other studies have also revealed that attitude and subjective norms evidently determined the intention for behaviors, such as consumption of amphetamines (28)and change or purchase of cars (29).

The results of regression analysis for prediction of behavior based on attitude, subjective norms, and behavioral intention showed that the prediction model was significant. In this model, subjective norms and behavioral intention significantly predicted and determined the students’ behavior. This result emphasizes the role of human factors as the key determinants of dressing behavior. However, attitude played no significant role in this regard (22). Overall, the roles of attitude and subjective norms in determination and prediction of behaviors are affected by various factors, including type of behavior, personal features, cultural differences, and situational factors (14, 22). A study on the citizens’ behaviors regarding consumption of water showed that the individuals’ behaviors were mostly controlled by their attitude and normative factors played no important roles in this regard (22). However, different results were obtained concerning prediction of condom use. Hence, the significant determining power of attitude for intention but not behavior might indicate that other factors, such as perceived behavioral control, self-efficacy, and social communication skills, overshadow the relationship between behavior and attitude (13). Considering the cultural factors, on the other hand, the students with different fields of study might have different subcultures. Situational factors, such as the college where one studies, may play a role, as well. In this study also, the students studying in different colleges had more or less different attitudes and subjective norms.

In general, individuals tend to interact with those with similar beliefs, attitudes, and information. Thus, peers play a major role in socialization of consumers of goods, such as clothes (30). Similarly, the results of some studies showed that in comparison to parents, peers played a more important role in the students’ type of dressing. Parents play the initial role in lower ages (30, 31). However, as the child grows up and enters social networks, such as school and friends, the role of parents is diminished and replaced by that of peers (30). Social influence is reflected in the individuals’ attitude, internalized group norms, perceived inter-personal pressure, and subjective norms (32).

The results of a study in Nigeria showed that dressing habits were impacted by similar factors in both sexes. In addition, both girl and boy adolescents’ way of dressing was affected by social approval, anxiety, modernity, and the tendency for showing off. The government, through schools and other community organizations, should make people familiar with appropriate dressing patterns (33).

In the study carried out in Malaysia, in comparison to male students, female ones were more affected by their peers in purchasing goods. This is due to the fact that girls are more sensitive to their physical appearance and their behaviors are more affected by their peers. The girls’ higher scores of subjective norms in this study can also demonstrate the higher importance of social preferences in selection of type of dressing for girls (30).

The findings of this study indicated that the students studying in different colleges had more or less different subjective norms. Also, differences were observed between the two sexes as well as between the students studying in different colleges regarding attitude and behavioral intention. Thus, various approaches and methods should be developed considering these differences. 

**Limitations**

Similar to many other studies which have employed the theory of reasoned action, the present study relied on the data obtained through self-report, while evidence has shown the probability of confounding of the data gathered using this method. 

## Conclusion

The findings of the present study demonstrated the efficiency of theory of reasoned action in analysis and determination of the students’ behavior as well as in induction of educational and cultural strategies regarding dressing and university dress code. According to the results, measures should be taken to increase the students’ perception and information regarding the content of university dress code. Moreover, considering the effectiveness of perceived social pressure and support (subjective norms), propagation of encouraging and motivating discussions among students, professors, and parents is of great importance. Finally, a scientific approach based on reliable findings obtained in theory-based studies and educational as well as cultural planning is recommended to be used to analyze the dressing behavior based on basic values and beliefs of an Islamic society. 
